# Supplementary material for: Regulation of somatic stem cell development through positional and proliferative signals during Drosophila melanogaster pupal ovary development resembles the framework governing adult stem cell behavior
Source: Genetics. 2026 May 12;233(2):iyag093. doi: 10.1093/genetics/iyag093 (PMC13291918; doi:10.1093/genetics/iyag093)
Supplement: iyag093_Supplementary_Data [file iyag093_Supplementary_Data.zip › FigureS2.pdf]

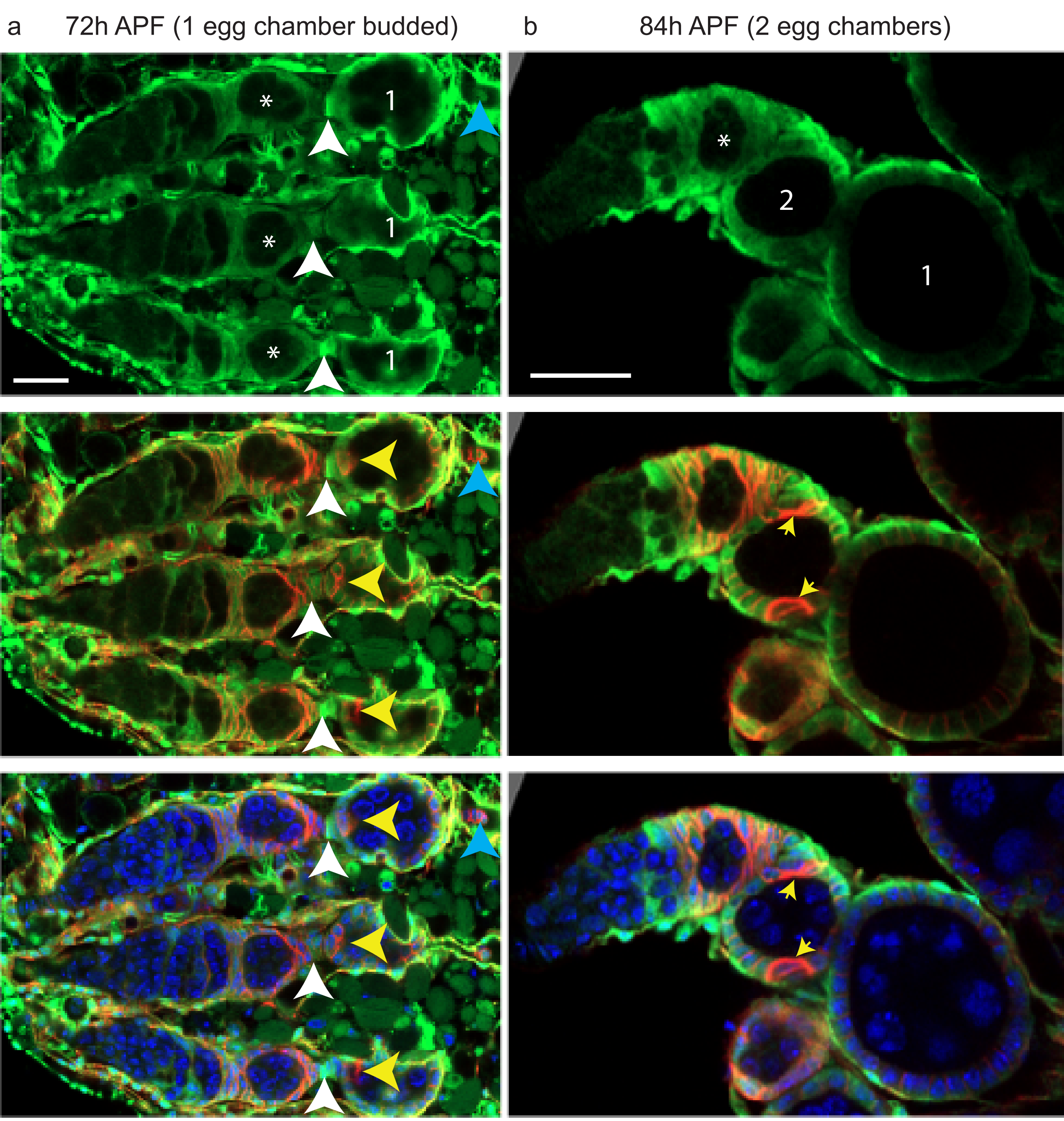


Figure S2: JAK/STAT signaling in pupal germaria after stalk and polar cell formation. Pupal ovaries expressing STAT-GFP were stained for GFP (green), Fasciclin 3 (red), and with DAPI (blue). Bars, 20μm. (a) Image from a pupal ovary at 72h APF, after the first egg chamber has budded (labeled with a 1), polar cells have formed on the first egg chamber (yellow arrowheads indicate the anterior polar cells), and a stalk has formed between the egg chamber and the germarium. Epithelial sheath cells that surround each developing ovariole and the entire ovary strongly express STAT-GFP. STAT-GFP is also expressed in follicle cells in the egg chamber and the germarium, and around the cysts just anterior to the Fas3 border. The most mature egg chamber in the germarium is marked by an asterisk. STAT-GFP is often prominent in stalk cells (white arrowheads) and is also detected in the basal stalk (blue arrowhead). (b) By 84h APF, two egg chambers have budded (labeled with 1 and 2), and STAT-GFP expression resembles the adult pattern, where it is expressed in follicle cells of the egg chambers and the posterior of the germarium, and tapers off anterior to the Fasciclin 3 border. Yellow arrowheads indicate the polar cells in the 2nd egg chamber and the asterisk marks the most mature egg chamber in the germarium.
