# Supplementary material for: Regulation of somatic stem cell development through positional and proliferative signals during Drosophila melanogaster pupal ovary development resembles the framework governing adult stem cell behavior
Source: Genetics. 2026 May 12;233(2):iyag093. doi: 10.1093/genetics/iyag093 (PMC13291918; doi:10.1093/genetics/iyag093)
Supplement: iyag093_Supplementary_Data [file iyag093_Supplementary_Data.zip › Supplemental_Figure_S1_GENETICS-2026-308979.pdf]

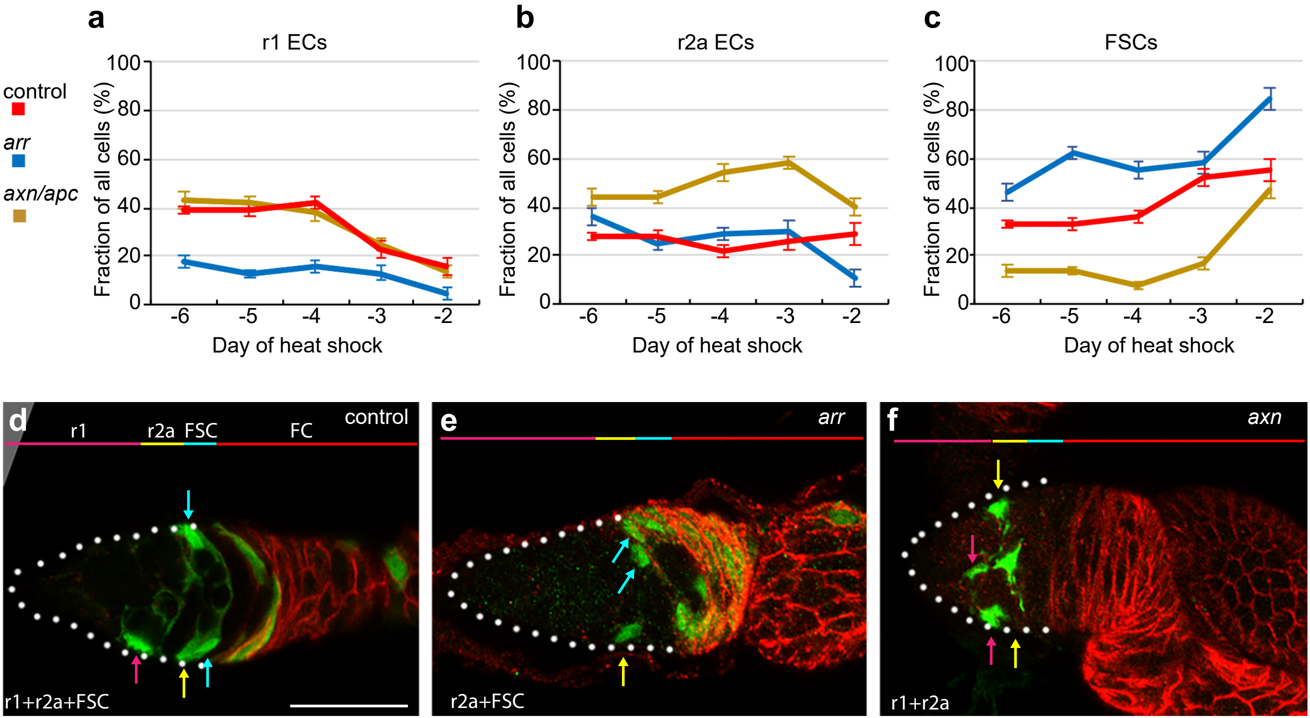

Figure S1: Wnt signaling influences over different periods of pupation. (a-f) MARCM lineages with genetically altered Wnt pathway activity were induced from 6d to 2d before eclosion and examined in 2d-old adults. (a-c) The total number of marked cells of each cell type (r1 or r2a ECs and FSCs) was counted over all samples and expressed as a percentage of all marked ECs and FSCs for control clones (red), *arr* mutant clones lacking Wnt pathway activity (blue) and *axn* or *apc* mutant clones (combined data, gold) with increased Wnt pathway activity. d-f) Images of germaria stained for Fas3 (red) show examples of a (d) control lineage with all cell types labeled, (e) an arr lineage with marked r2a ECs, FSCs and FCs (f) an *axn* lineage with only marked r1 and r2a ECs. Scale Bar, 20 μm. White dotted line shows outline of germaria.
