# Supplementary material for: Regulation of somatic stem cell development through positional and proliferative signals during Drosophila melanogaster pupal ovary development resembles the framework governing adult stem cell behavior
Source: Genetics. 2026 May 12;233(2):iyag093. doi: 10.1093/genetics/iyag093 (PMC13291918; doi:10.1093/genetics/iyag093)
Supplement: iyag093_Supplementary_Data [file iyag093_Supplementary_Data.zip › Supplemental_Figure_S3_GENETICS-2026-308979.pdf]

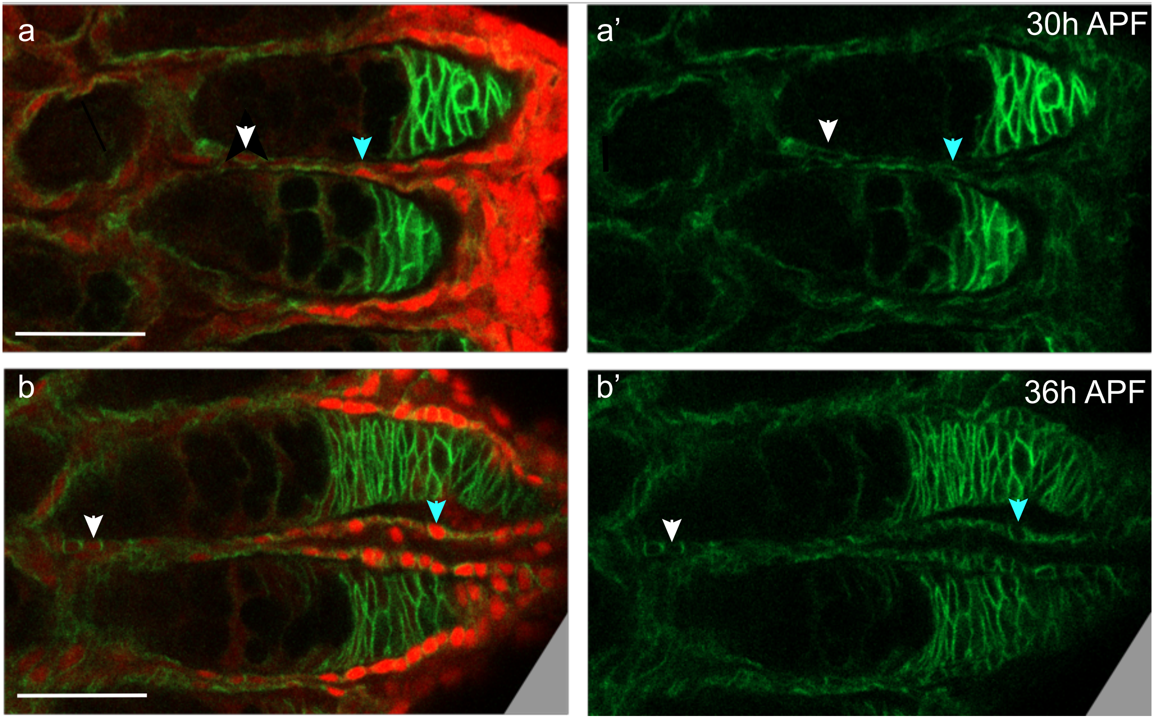

Figure S3: STAT-RFP expression in epithelial sheath cells. (a,a') Fasciclin 3 (green) expression is first detected in epithelial sheath cells of the pupal ovary starting around 30h APF. STAT activity, as shown by STAT-RFP expression (red), also begins to be detected in the epithelial sheath around 30h and is stronger in cells on the basal/posterior side of the epithelial sheath (cyan arrowheads) than in cells surrounding the anterior half of the germarium (white arrowheads). (b, b') A 36h APF ovary shows similar patterns of Fasciclin 3 expression on epithelial sheath cell membranes and STAT-RFP in the sheath cells, with stronger expression posterior (cyan arrowheads) than anterior (white arrowheads). Scale Bars, 20 μm.
