# Supplementary material for: Regulation of somatic stem cell development through positional and proliferative signals during Drosophila melanogaster pupal ovary development resembles the framework governing adult stem cell behavior
Source: Genetics. 2026 May 12;233(2):iyag093. doi: 10.1093/genetics/iyag093 (PMC13291918; doi:10.1093/genetics/iyag093)
Supplement: iyag093_Supplementary_Data [file iyag093_Supplementary_Data.zip › Supplemental_Video_S1_legend_GENETICS-2026-308979.docx]

Supplementary Video 1.

Fz3-RFP expression in a 27h APF ovary. In developing germaria, Fz3-RFP (red) is expressed in somatic precursors in a domain beginning adjacent to Cap cells and ending with a posterior decline to undetectable levels at the location where precursors express Fas3 (white). Nuclei are stained with DAPI (blue) and anterior/apical is at the top of the image. The video shows a succession of z-planes through the tissue.
