# Supplementary material for: Regulation of somatic stem cell development through positional and proliferative signals during Drosophila melanogaster pupal ovary development resembles the framework governing adult stem cell behavior
Source: Genetics. 2026 May 12;233(2):iyag093. doi: 10.1093/genetics/iyag093 (PMC13291918; doi:10.1093/genetics/iyag093)
Supplement: iyag093_Supplementary_Data [file iyag093_Supplementary_Data.zip › Supplementary_File_1_GENETICS-2026-308979/Supplementary File 1 Explanation of spreadsheets.docx]

**Supplementary File 1. Supplementary spreadsheets with raw data and calculations underlying all presented data**

Sets of spreadsheets are arranged in folders, as described below.

**A. “Full Data with Significance” folder**

**Single-cell 0hAPF calculations**

All summary clone data for lineages induced at 0h APF together with the calculations converting data to estimations of single-cell lineage data and averaging of all control values and other genotypes tested in more than one experiment.

**Single-cell -2dAPF calculations**

All summary clone data for lineages induced at -2d APF together with the calculations converting data to estimations of single-cell lineage data and averaging of all control values and other genotypes tested in more than one experiment.

**0h APF Significance Fisher** and **-2d APF Significance Fisher**

Copies of the two previous spreadsheets (numbers only, no formulae exposing the calculation methods) with additional columns to show p values of significant differences to controls if p<0.05 using Fisher’s exact two-tailed test.

**0h APF Controls Variation**

Calculation of SD and SEM for single-cell lineage parameters among control experiments and comparison to expectations from probabilistic labeling of precursors in different A/P locations.

**B. “GraphsRawData” folder**

**0h APF Graphs Fig1,3**

First sheet is 0h APF master sheet (from the single-cell calculation sheet). Others show data for Figs. 1b-d and Fig. 3i.

**0h APF Graphs Fig4-10**

First sheet is 0h APF master sheet (from the single-cell calculation sheet). Others show data for Figs. 4, 6, 8h, 9d, 10g

**-2d APF Graphs**

First sheet is -2d APF master sheet (from the single-cell calculation sheet). Others show data for Figs. 3j, 8i, 9e.

**0hAPF Results-orderedCons**

Calculations for Fig. 1b-d and Fig. 3f.

**-3.5d axn August 2023 Numbers**

Control and *axn* lineages initiated 3.5d before eclosion (36h APF); raw data and deduced frequencies of clone types.

**-4d arr DIAP Numbers**

Control and *arr; UAS-DIAP1* lineages initiated 4d before eclosion (24h APF); raw data and deduced frequencies of clone types.

**Fig. S1 Graph Data**

Data for Supplementary Fig. 1.

**Fig5 Fz3RFP Graph**

Data for Fig. 5 graph of Fz3-RFP Wnt reporter intensities over the germarium at different times.

**-3.5d stat November 2021 Numbers**

*stat* lineages initiated 3.5d before eclosion (36h APF); raw data and deduced frequencies of clone types.

**C. “Cell Division Data” folder**

**FC occupancy aggregate**

Raw data and aggregation for fraction of terminal egg chamber occupied by marked FCs and frequency of additional FC labeling in penultimate or more anterior egg chambers for cycE and cutlet genotype lineages (initiated at 0h APF and -2d APF, leading to graph in Fig. 3f), and for selected Hedgehog and Yorkie genotypes (0h APF Hh Yki TermFC). Raw cell number data for EC-only and EC/FSC lineages of select Hh Yki genotypes to estimate SEM (Cell#). Summary data for fraction of available cysts with marked FCs in FSC-containing lineages (%cystsMarkedFCs 0h APF).

**Division Measures Hh/Yki**

Summary data for graphs in Fig. 10h-k.

**D. “Final Raw Data Spreadsheets” folder**

Raw data lineage spreadsheets:

“**Compilation of Numbers**” named by Figure and time of lineage initiation (“-5d” = 0h APF, “-7d” = -2d APF).

First sheet in a pair is the raw tabulation of marked cells for each sample of a given genotype. The second sheet compiles the raw data to give a variety of measures, including those transferred to the master spreadsheets used to estimate single-cell lineage parameters (“calculations” spreadsheets).

**“FCNumberPosteriorSpreadsheets”**

Raw data of terminal egg chamber occupancy by marked FCs for seven named experiments.

**“Spreadsheets with cyst occupancy counts”**

Raw data of egg chamber occupancy by marked FCs for twelve named experiments.
